# Dose-dependent effect of lamotrigine on quetiapine serum concentration in patients using instant release tablets

**DOI:** 10.1007/s00228-024-03655-z

**Published:** 2024-02-23

**Authors:** Kristine Hole, Silje K Lorentsen, Karoline L Nordby, Marie Slettvik, Ida TG Sørum, Espen Molden, Tore Haslemo

**Affiliations:** 1 Center for Psychopharmacology, Diakonhjemmet Hospital, Oslo, Norway; 2 Department of Life Sciences and Health, Oslo Metropolitan University, Oslo, Norway; 3 Department of Pharmaceutical Biosciences, School of Pharmacy, University of Oslo, Oslo, Norway

**Keywords:** Drug-drug-interaction, Lamotrigine, Pharmacokinetic variability, Quetiapine

## Abstract

**Purpose:**

Lamotrigine was previously reported to reduce serum concentration of quetiapine. The aim of this study was to investigate whether lamotrigine dose or quetiapine formulation was of importance for the drug interaction.

**Methods:**

Patients combining lamotrigine with quetiapine (cases) were included retrospectively from a routine therapeutic drug monitoring (TDM) service, as were a control group of patients using quetiapine without any interacting drugs. The case and control groups were divided into groups using immediate release (IR) and extended release (XR) quetiapine. The case group was further split into high-dose (> 200 mg/day) and low-dose (≤ 200 mg/day) lamotrigine users. Quetiapine concentration-to-dose (C/D) ratio and metabolite-to-parent ratio (MPR) were compared between the control group and dose-separated case groups using ANOVA test and *t*-tests.

**Results:**

In total, 406 patients were included. The mean C/D ratio of IR quetiapine was 46% lower in the high-dose lamotrigine group compared with the control group (*P* < 0.001), while no interaction effect was present in the low dose lamotrigine group (*P* = 0.7). Regardless of lamotrigine dose, there was no difference in quetiapine C/D ratio for patients using the XR formulation (*P* = 0.4). The quetiapine MPR was unaffected regardless of formulation and lamotrigine dose (*P* ≥ 0.06).

**Conclusion:**

The effect of lamotrigine in reducing quetiapine concentration is only significant for patients using quetiapine IR tablets who are treated with lamotrigine doses > 200 mg/day. Because of high variability in the interaction effect, TDM of quetiapine should be recommended during co-prescription of high-dose lamotrigine.

## Introduction

Quetiapine is an atypical antipsychotic drug frequently used in treatment of schizophrenia and bipolar disorder [1]. Individual variability in quetiapine pharmacokinetics is extensive, which may reflect interpatient differences in quetiapine metabolism [2, 3]. Cytochrome P450 (CYP) 3A4 is considered the primary enzyme in the metabolic clearance of quetiapine and the main enzyme in the formation of the pharmacologically active metabolite N-desalkylquetiapine [4]. Other enzymes, such as CYP2D6 and UDP-glucuronosyltransferase (UGT) enzymes, also contribute to quetiapine metabolism, albeit minorly [2]. The therapeutic doses of quetiapine range from 150 to 800 mg daily, and the therapeutic reference range is defined as 260–1300 nmol/L (100–500 ng/mL) [5]. The consensus guidelines for therapeutic drug monitoring (TDM) in neuropsychopharmacology recommend TDM of quetiapine for dose titration and for special indications or problem solving [5]. The tolerability of quetiapine is quite good, but insufficient mood stabilizing or antipsychotic effect due to low concentrations may be deleterious.

The antiseizure medication lamotrigine is also approved for bipolar disorder, and co-prescription with quetiapine is common [1]. A potential drug-drug interaction between lamotrigine and quetiapine has previously been reported from two TDM services [6, 7]. The first study investigated the effect of age, sex, and various comedications on the pharmacokinetics of quetiapine using linear mixed model analysis. They reported a 17% lower quetiapine serum concentration in 147 TDM samples from patients co-medicated with lamotrigine compared with 1123 TDM samples from patients using no co-medication, but the interaction was not considered to be of clinical relevance [6]. The second study reported that lamotrigine use was associated with a 58% reduction in quetiapine concentration-to-dose (C/D) ratio, but only 22 lamotrigine-treated patients and 22 matched controls were included [7]. The authors suggested that the underlying interaction mechanism could possibly be lamotrigine induction of quetiapine glucuronidation [7]. However, neither of the studies investigated the potential impact of lamotrigine dose or quetiapine formulation—i.e., immediate release (IR) or extended release (XR) tablets—on the reduced serum concentration of quetiapine.

The aim of the present study was to investigate to what extent lamotrigine dose and quetiapine formulation are of importance for the previously described drug-drug interaction between these agents.

## Methods

### Patient inclusion

Patients combining lamotrigine with quetiapine (cases) were included retrospectively from a routine TDM service at Center for Psychopharmacology, Diakonhjemmet Hospital, as was a control group of patients who according to their TDM request forms used quetiapine without any interacting drugs. The case and control groups were divided into groups using immediate release (IR) and extended release (XR) quetiapine. The case group was further split into high-dose (> 200 mg/day) and low-dose (≤ 200 mg/day) lamotrigine users.

The case group was included during the time period 2009 to 2022 if the TDM request forms contained information about quetiapine formulation and daily dose of both drugs. To include a control group of approximately the same size as the case group, patients monitoring quetiapine without concomitant use of lamotrigine were included from 2021 to 2022 if the TDM request forms contained information about quetiapine dose and formulation. Patients were included only if the time between last drug intake and sampling was between 6 and 24 h for quetiapine IR tablets, 12 and 16 h for quetiapine XR tablets, and between 10 and 30 h for lamotrigine. We allowed a wide time interval for inclusion of quetiapine IR tablets because these samples were converted to the 12-h concentration before statistical analysis (this is described in detail under data analysis). If more than one TDM measurement was available for a patient, only the last measurement was included.

Samples were excluded (a) if the patient used less than 150 mg quetiapine daily, (b) if levels of quetiapine or lamotrigine were undetectable, (c) if the patient used quetiapine IR and XR tablets concurrently, (d) if the TDM requisition forms mentioned that the patient used any CYP-interacting drugs according to a compilation from Hiemke et al. [5], and (e) if patient age was less than 18 yrs or more than 64 yrs. Observed interacting drugs included bupropion, carbamazepine, duloxetine, esomeprazole, fluoxetine, fluvoxamine, levomepromazine, paroxetine, and valproic acid.

### Ethical considerations

The use of pseudonymized patient data for the purpose of this study was approved by the Regional Committee for Medical and Health Research Ethics (ref. 452249) and the Diakonhjemmet Hospital Investigational Review Board. Since the study was based on existing data retrospectively retrieved from a routine TDM service, ethical approval was granted without the requirement of patient consent.

### Serum concentration measurements

Serum concentration measurements of quetiapine, N-desalkylquetiapine, and lamotrigine were performed by validated analytical methods developed for routine TDM analyses at Center for Psychopharmacology. During the time span of the TDM analyses of the serum samples, the analytical assays were modified due to renewal of analytical instruments, and all modifications were cross-validated. The current analyses were based on ultrahigh-performance liquid chromatography (UPLC) high-resolution mass spectrometry (HRMS). Serum samples were prepared by protein precipitation using a 9:1 acetonitrile:methanol mixture which included the internal standards quetiapine-^13^C_4_, N-desalkylquetiapine-D_8_, and lamotrigine-^13^C_7_,^15^N. Purified samples were injected into a Vanquish-UPLC system (Thermo Fisher Scientific, Waltham, MA, USA). Chromatographic separation was achieved with an Xbridge BEH C18 column (2.6 µm, 2.1 × 75 mm; Waters Corporation, Milford, MA, USA) kept at 35 °C. The mobile phase was composed of ammonium acetate buffer (pH 4.8) and acetonitrile (20–52%). Retention times were 1.84 min for quetiapine, 1.70 min for N-desalkylquetiapine, and 0.97 min for lamotrigine. Detection was achieved with Q Exactive Orbitrap high resolution MS (Thermo Fisher Scientific) operating in positive mode. The m/z values were 384.17402 for quetiapine, 296.12159 for N-desalkylquetiapine, and 256.01513 for lamotrigine. Lower and upper limits of quantification were 20–2400 nmol/L (8–920 ng/mL) for quetiapine, 25–3000 nmol/L (7–885 ng/mL) for N-desalkylquetiapine, and 2–70 µmol/L (0.5–18 µg/mL) for lamotrigine. Inter- and intra-day imprecision and inaccuracy parameters of the assay were < 15%.

### Data analysis

Quetiapine IR and XR tablets have different pharmacokinetic profiles. For example, the time to maximum concentration is 2 h for IR tablets and 5 h for XR tablets, and the serum concentration of XR tablets is reported to be almost twice the level of IR tablets at 12 h [8]. Therefore, the interaction and control groups were compared separately for IR and XR quetiapine. For XR quetiapine, a constant serum concentration was assumed between 12 and 16 h [8], and the measured serum concentration was used for calculation of both concentration-to-dose (C/D) ratio and metabolite-to-parent ratio (MPR).

To reduce the influence of unequal sampling time for IR tablets, the measured serum concentration at *t* hours since last dose intake was converted to a 12-h concentration (*C*_*12*_) according to the following equation: ${C}_{12}={C}_{t}{e}^{-k\left(12-t\right)}$

The elimination constant *k* was calculated from the equation $k= \frac{ln2}{{t}_{\nicefrac{1}{2}}}$, with a half-life (*t*_1/2_) of 7 h [2]. The *C*_*12*_ was used to calculate C/D ratio for IR quetiapine, but not for calculation of MPR. That is, all C/D ratios we report for IR quetiapine are based on the calculated *C*_*12*_.

Comparison of sex distribution between groups was performed with chi-square test. Comparison of age, dose, and sampling time was compared using one-way ANOVA tests. Quetiapine C/D ratio and MPR were compared between the control group and the low-dose (≤ 200 mg) and high-dose (> 200 mg) lamotrigine groups with one-way ANOVA and two-sided *t*-tests. Finally, Spearman correlation was used to investigate a potential association between quetiapine C/D ratio and lamotrigine dose or serum concentration. *P* < 0.05 was considered statistically significant. Statistical analyses were performed using IBM SPSS Statistics for Windows, Version 28.0 (Armonk, NY: IBM Corp), and GraphPad Prism version 10.0.0 for Windows (GraphPad Software, Boston, MA, USA) was used for graphical presentations.

## Results

### Study population

**Fig. 1:**
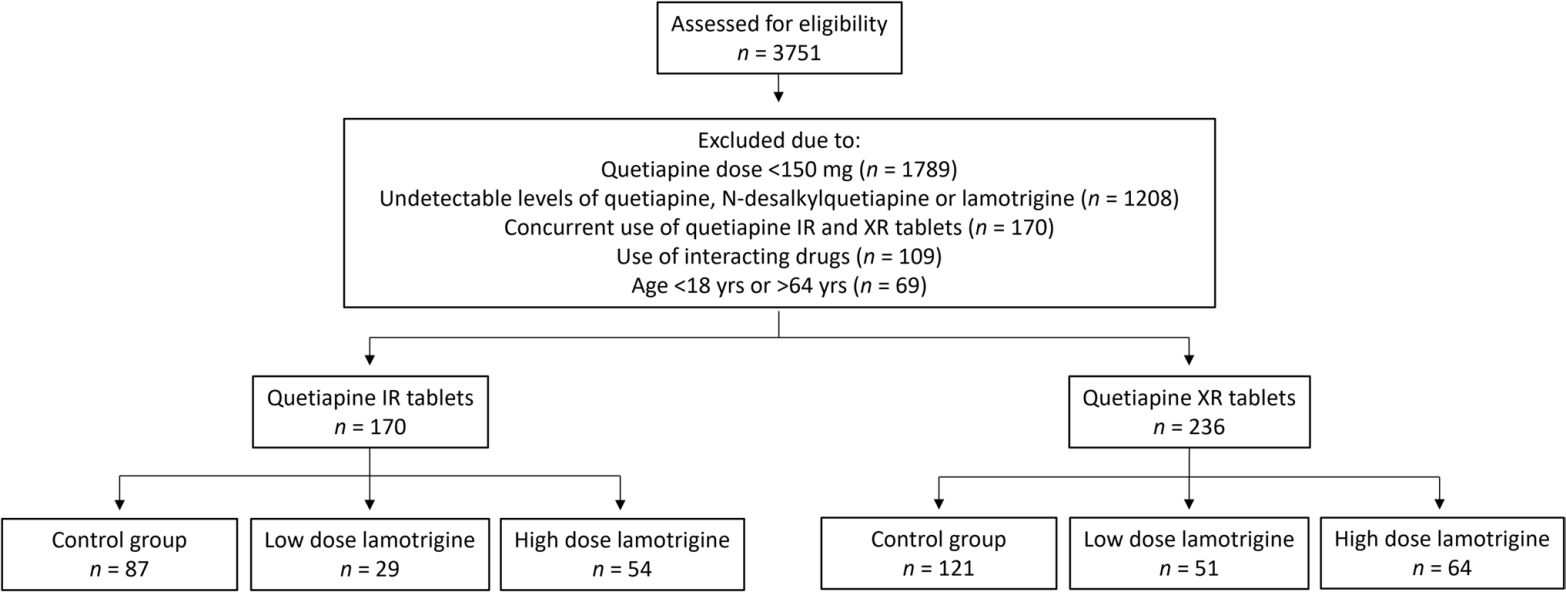
Flowchart of patient inclusion and exclusion

At first, 3751 patients were eligible for inclusion in the study. Inclusion and exclusion are presented in Fig. 1. After exclusion, there were 170 patients included who used quetiapine IR tablets and 236 patients who used XR tablets. These groups were further divided into a control group and two case groups, i.e., a low-dose lamotrigine group (≤ 200 mg/day) and a high-dose lamotrigine group (> 200 mg/day). The subgrouping was similar for both quetiapine IR and XR tablets (Fig. 1).

Patient characteristics are presented in Table 1. For XR quetiapine, the low-dose and high-dose lamotrigine groups comprised 77% and 80% women, respectively, compared with 59% women in the control group (*P* = 0.005). These sex difference in lamotrigine users vs. non-users was not present for patients treated with IR quetiapine (*P* = 0.7).

**Table 1:** Patient characteristics

	Quetiapine immediate release				Quetiapine extended release			
	Control group (*n* = 87)	Low-dose lamotrigine (0–200 mg) (*n* = 29)	High-dose lamotrigine (> 200 mg) (*n* = 54)	*P*	Control group (*n* = 121)	Low-dose lamotrigine (0–200 mg) (*n* = 51)	High-dose lamotrigine (> 200 mg) (*n* = 64)	*P*
Women, *n* (%)	54 (62)	20 (69)	37 (69)	0.7	71 (59)	39 (77)	51 (80)	0.005
Age (y)	43 (40; 46)	41 (36; 46)	38 (35; 41)	0.1	42 (39; 44)	39 (36; 43)	42 (39; 45)	0.5
Quetiapine dose (mg)	398 (343; 453)	347 (263; 430)	367 (317; 417)	0.5	449 (403; 494)	408 (344; 472)	380 (328; 433)	0.2
Quetiapine sampling time (h)	13.5 (12.9; 14.1)	12.8 (12.1; 13.5)	12.8 (12.5; 13.2)	0.2	13.4 (13.2; 13.7)	13.4 (13.0; 13.8)	13.4 (13.0; 13.7)	0.9

Values are presented as mean (95% confidence interval). *P* values are derived from chi-square and ANOVA tests

### Effect of lamotrigine on immediate-release quetiapine

**Fig. 2:**
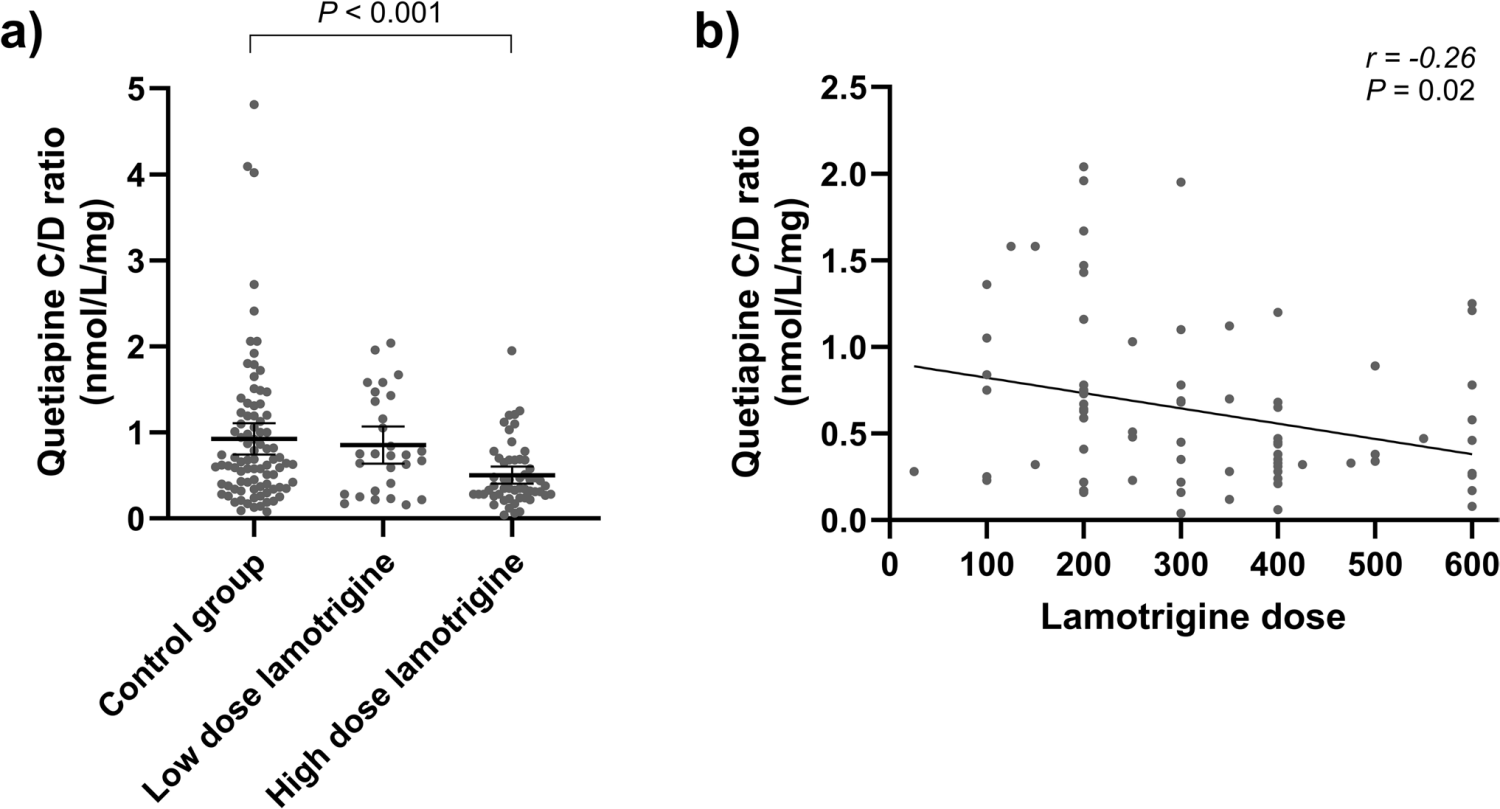
**a** Scatterplot of instant release quetiapine concentration-to-dose (CD) ratio in the control group of patients not co-medicated with lamotrigine, in the low-dose lamotrigine group (≤ 200 mg/day) and in the high-dose lamotrigine group (> 200 mg/day). Solid lines illustrate the mean values and the error bars illustrate the 95% confidence intervals. **b** Spearman correlation of quetiapine C/D ratio and lamotrigine dose. Linear regression line is added for visual purposes

The quetiapine C/D ratio based on serum concentrations adjusted to *C*_*12*_ ranged 120-fold among all patients using IR tablets regardless of comedication, from 0.04 to 4.81 nmol/L/mg. Mean quetiapine C/D ratios and MPRs in the control group vs. the low-dose and high-dose lamotrigine groups are presented in Table 2. The patients using high-dose lamotrigine had 46% lower mean quetiapine C/D ratio than the control group (*t*-test *P* < 0.001, Fig. 2a) and 41% lower quetiapine C/D ratio than the low-dose lamotrigine group (*P* = 0.001). There was no difference in quetiapine C/D ratio between the low dose lamotrigine group and the control group (*P* = 0.7). We observed a weak negative correlation between quetiapine C/D ratio and lamotrigine dose (Spearman’s *r* =  − 0.26, *P* = 0.02, Fig. 2b), but not between quetiapine C/D ratio and lamotrigine concentration (*r* =  − 0.06, *P* = 0.6). The mean quetiapine MPR was borderline significantly higher by 38% in the high-dose lamotrigine group compared with the control group (*P* = 0.06), but there was no difference in MPR between the low-dose lamotrigine group and the control group (*P* = 0.8).

**Table 2:** Quetiapine concentration-to-dose (C/D) ratio and metabolite-to-parent ratio (MPR) in control and case groups

	Quetiapine immediate release				Quetiapine extended release			
	Control group (*n* = 87)	Low-dose lamotrigine (0–200 mg) (*n* = 29)	High-dose lamotrigine (> 200 mg) (*n* = 54)	*P*	Control group (*n* = 121)	Low-dose lamotrigine (0–200 mg) (*n* = 51)	High-dose lamotrigine (> 200 mg) (*n* = 64)	*P*
Quetiapine C/D ratio (nmol/L/mg)	0.92 (0.74; 1.11)	0.85 (0.64; 1.07)	0.50 (0.40; 0.60)	0.002	1.18 (0.96; 1.40)	1.16 (0.92; 1.41)	0.95 (0.78; 1.13)	0.4
Quetiapine MPR	2.37 (1.77; 2.96)	2.25 (1.58; 2.92)	3.28 (2.56; 4.01)	0.09	1.73 (1.45; 2.01)	1.49 (1.22; 1.76)	1.85 (1.48; 2.22)	0.4

Values are presented as mean (95% confidence interval). *P* values are derived from one-way ANOVA tests

### Effect of lamotrigine on extended-release quetiapine

The quetiapine C/D ratio ranged 190-fold among all patients using XR tablets regardless of comedication, from 0.05 to 9.52 nmol/L/mg. Mean quetiapine C/D ratios and MPRs in the control group vs the low-dose and high-dose lamotrigine groups are presented in Table 2 and did not significantly differ between the groups (*P* = 0.4). There was no correlation between quetiapine C/D ratio and lamotrigine dose or serum concentration (*P* ≥ 0.2).

## Discussion

In the present study, we show that the interaction between lamotrigine and quetiapine is dependent on lamotrigine dose and quetiapine tablet formulation. Patients using IR quetiapine are at risk of lamotrigine interaction, and among these, the average quetiapine C/D ratio was 46% lower in patients co-medicated with high-dose lamotrigine compared with the control group without lamotrigine. Furthermore, IR quetiapine C/D ratio was negatively correlated with lamotrigine dose. The dose-dependent interaction indicates that patients may require dose increase of IR quetiapine with co-prescription of high-dose lamotrigine. We did not observe an interaction between lamotrigine and quetiapine XR tablets.

Previously, Andersson et al. reported 58% lower quetiapine C/D ratio in lamotrigine patients (*n* = 22) compared with a matched control group (*n* = 22), and hypothesized that lamotrigine induced glucuronidation of quetiapine [7]. They did not report lamotrigine doses of the included patients, but 86% of the patients used IR quetiapine [7]. Castberg et al. have reported that quetiapine C/D ratio was 17% lower with lamotrigine use (*n* = 147) compared with serum samples from patients not using co-mediation (*n* = 1123) [6]. This study was performed before the introduction of quetiapine XR tablets, but lamotrigine dose of the included patients was not reported [6]. It is possible that the discrepancy in reported interaction effect is due to differences in lamotrigine doses between the two studies, since the present study showed that only high-dose lamotrigine was associated with lower quetiapine C/D ratio, not low-dose lamotrigine.

The mechanism of the drug-drug interaction is not yet elucidated. Lamotrigine is reported to exhibit a modest dose-dependent induction of its own metabolism, probably via uridine glucuronosyl transferase (UGT) enzymes [9]. The glucuronidation pathway for quetiapine is not well described [2], but a study in rats recently demonstrated that UGT inhibition led to 53% elevation of quetiapine exposure, supporting that UGT enzymes contribute to quetiapine metabolism [10]. Therefore, lamotrigine induction of quetiapine glucuronidation is a possible mechanism for the observed interaction. The fact that lamotrigine in the present study showed no significant effect on the *N*-desalkylquetiapine-to-quetiapine metabolic ratio, which mainly reflect CYP3A4 metabolism, indicates that lamotrigine is not an inducer of CYP3A4.

We did not detect any correlation between quetiapine C/D ratio and lamotrigine serum concentration, only with lamotrigine dose. This may suggest that the interaction mechanism is related to a change in pharmacokinetics of the absorption process of quetiapine, which coincide with the interaction effect being restricted to the quetiapine IR formulation, and not to quetiapine XR. The fact that quetiapine C/D ratio was associated with pre-systemic exposure (= dose) and not systemic lamotrigine exposure (= measured venous serum concentration), supports that the interaction is related to changes in quetiapine absorption in the intestine. Similarly, Andersson et al*.* did not detect a correlation between lamotrigine exposure and quetiapine C/D-ratio [7]. However, the present evidence is far from conclusive on the mechanism underlying the interaction between lamotrigine and quetiapine, which should be investigated in further studies, preferably by in vitro experiments or gene expression analysis, to elucidate potential metabolic pathways.

Quetiapine displayed considerable interindividual variability in C/D ratio, with 120-fold variation for IR tablets—where the C/D ratio was calculated from *C*_12_—and 190-fold variation for XR tablets. Previous studies have also reported great variation in both intra- and interindividual serum concentrations of quetiapine [2, 11–13]. Due to a short half-life of 7 h and an extreme variability in serum concentration peaks and throughs, it is difficult to eliminate variability due to different sampling times. For IR tablets, we calculated through concentration for 12 h post-dose to reduce sampling time variability. For XR tablets, however, we assumed stabile serum concentrations between 12 and 16 h post-dose, as reported by Figueroa et al. [8].

Due to the naturalistic nature of the study, an important limitation is uncertainty regarding the adherence of included patients. An increased level of nonadherence with prescription of more than one drug is possible. To reduce the chances of poor compliance among the study population, we excluded patients with nondetectable levels of quetiapine, which may reflect noncompliance. Furthermore, we did not include patients using < 150 mg quetiapine daily, to avoid intermittent quetiapine use for off-label indications such as sleeping aid. We also excluded patients < 18 yrs and > 64 yrs to avoid age-related variation in quetiapine C/D ratio [6, 12]. Another study limitation was that we lacked information such as diagnosis, comorbidity, organ function, and concurrently used drugs not provided on the TDM request forms. Therefore, we cannot rule out that some of the included patients used drugs other than lamotrigine which may have interacted with quetiapine. These factors may have contributed to the great variability observed in quetiapine C/D ratio. Unfortunately, the lack of clinical data in this study also extended to information regarding therapeutic efficacy and side effects. Thus, it was not possible to investigate whether the reduced quetiapine levels were associated with clinical consequences such as therapeutic failure. The clinical consequences of the drug-drug interaction is unclear and should be addressed in prospective clinical studies. Furthermore, studies incorporating long-term monitoring and population pharmacokinetic modelling could add to the understanding of the interaction and contribute personalized treatment plans for affected patients.

## Conclusion

The present study shows that the interaction between lamotrigine and quetiapine is dependent on lamotrigine dose and quetiapine tablet formulation. For patients using IR quetiapine, high-dose lamotrigine was associated with 46% lower quetiapine concentration than patients not co-medicated with lamotrigine. Furthermore, lamotrigine dose was negatively correlated with the C/D ratio of IR quetiapine. The dose-dependent interaction indicates that patients may require dose-increase of IR quetiapine with co-prescription of high-dose lamotrigine. However, because of high variability in interaction effect, we recommend TDM of quetiapine to ensure sufficient dosing and serum concentration during co-prescription of high-dose lamotrigine. We did not observe an interaction between lamotrigine and quetiapine XR tablets. The mechanism underlying the interaction should be investigated in further studies.

## Data Availability

Data that support the findings of this study are available on request from the corresponding author. The data are not publicly available due to privacy or ethical restrictions.

## References

[1] Nierenberg AA (2023). Diagnosis and treatment of bipolar disorder: a review. JAMA.

[2] DeVane CL, Nemeroff CB (2001). Clinical pharmacokinetics of quetiapine: an atypical antipsychotic. Clin Pharmacokinet.

[3] Mauri MC (2018). Clinical pharmacokinetics of atypical antipsychotics: an update. Clin Pharmacokinet.

[4] Grimm SW (2006). Effects of cytochrome P450 3A modulators ketoconazole and carbamazepine on quetiapine pharmacokinetics. Br J Clin Pharmacol.

[5] Hiemke C (2018). Consensus guidelines for therapeutic drug monitoring in neuropsychopharmacology: update 2017. Pharmacopsychiatry.

[6] Castberg I, Skogvoll E, Spigset O (2007). Quetiapine and drug interactions: evidence from a routine therapeutic drug monitoring service. J Clin Psychiatry.

[7] Andersson ML, Björkhem-Bergman L, Lindh JD (2011). Possible drug-drug interaction between quetiapine and lamotrigine–evidence from a Swedish TDM database. Br J Clin Pharmacol.

[8] Figueroa C (2009). Pharmacokinetic profiles of extended release quetiapine fumarate compared with quetiapine immediate release. Prog Neuropsychopharmacol Biol Psychiatry.

[9] GlaxoSmithKline AS Lamictal summary of product characteristics. Available from: https://www.legemiddelsok.no/. Accessed 4 Aug 2023

[10] Sattar H (2020). Role of glucuronidation pathway in quetiapine metabolism: an in vivo drug-drug interaction study between quetiapine and probenecid. Saudi J Med Med Sci.

[11] Hasselstrøm J, Linnet K (2004). Quetiapine serum concentrations in psychiatric patients: the influence of comedication. Ther Drug Monit.

[12] Solhaug V (2023). Impact of age, sex and cytochrome P450 genotype on quetiapine and N-desalkylquetiapine serum concentrations: a study based on real-world data from 8118 patients. Br J Clin Pharmacol.

[13] Jönsson AK, Spigset O, Reis M (2019). A compilation of serum concentrations of 12 antipsychotic drugs in a therapeutic drug monitoring setting. Ther Drug Monit.

